# Bacteriophage-mediated biosynthesis of MnO_2_NPs and MgONPs and their role in the protection of plants from bacterial pathogens

**DOI:** 10.3389/fmicb.2023.1193206

**Published:** 2023-06-15

**Authors:** Solabomi Olaitan Ogunyemi, Yasmine Abdallah, Ezzeldin Ibrahim, Yang Zhang, Ji’an Bi, Fang Wang, Temoor Ahmed, Dalal Hussien M. Alkhalifah, Wael N. Hozzein, Chengqi Yan, Bin Li, Lihui Xu

**Affiliations:** ^1^State Key Laboratory of Rice Biology and Breeding, Ministry of Agriculture Key Lab of Molecular Biology of Crop Pathogens and Insects, Institute of Biotechnology, Zhejiang University, Hangzhou, China; ^2^Plant Pathology Department, Faculty of Agriculture, Minia University, Elminya, Egypt; ^3^Institute of Biotechnology, Ningbo Academy of Agricultural Sciences, Ningbo, China; ^4^Department of Biology, College of Science, Princess Nourah Bint Abdulrahman University, Riyadh, Saudi Arabia; ^5^Botany and Microbiology Department, Faculty of Science, Beni-Suef University, Beni-Suef, Egypt; ^6^Institute of Eco-Environmental Protection, Shanghai Academy of Agricultural Sciences, Shanghai, China

**Keywords:** nanoparticles, bacteriophage, chlorophyll fluorescence, bacterial leaf blight, phytotoxicity, biosynthesis, Arabidopsis

## Abstract

**Introduction:**

*Xanthomonas* oryzae pv. *oryzae* (Xoo) is the plant pathogen of Bacterial Leaf Blight (BLB), which causes yield loss in rice.

**Methods:**

In this study, the lysate of Xoo bacteriophage X3 was used to mediate the bio-synthesis of MgO and MnO_2_. The physiochemical features of MgONPs and MnO_2_NPs were observed via Ultraviolet - Visible spectroscopy (UV–Vis), X-ray diffraction (XRD), Transmission/Scanning electron microscopy (TEM/SEM), Energy dispersive spectrum (EDS), and Fourier-transform infrared spectrum (FTIR). The impact of nanoparticles on plant growth and bacterial leaf blight disease were evaluated. Chlorophyll fluorescence was used to determine whether the nanoparticles application were toxic to the plants.

**Results:**

An absorption peak of 215 and 230 nm for MgO and MnO_2_, respectively, confirmed nanoparticle formation via UV–Vis. The crystalline nature of the nanoparticles was detected by the analysis of XRD. Bacteriological tests indicated that MgONPs and MnO_2_NPs sized 12.5 and 9.8 nm, respectively, had strong *in vitro* antibacterial effects on rice bacterial blight pathogen, Xoo. MnO_2_NPs were found to have the most significant antagonist effect on nutrient agar plates, while MgONPs had the most significant impact on bacterial growth in nutrient broth and on cellular efflux. Furthermore, no toxicity to plants was observed for MgONPs and MnO_2_NPs, indeed, MgONPs at 200 μg/mL significantly increased the quantum efficiency of PSII photochemistry on the model plant, Arabidopsis, in light (ΦPSII) compared to other interactions. Additionally, significant suppression of BLB was noted in rice seedlings amended with the synthesized MgONPs and MnO_2_NPs. MnO_2_NPs showed promotion of plant growth in the presence of Xoo compared to MgONPs.

**Conclusion:**

An effective alternative for the biological production of MgONPs and MnO_2_NPs was reported, which serves as an effective substitute to control plant bacterial disease with no phytotoxic effect.

## 1. Introduction

Bacterial leaf blight (BLB) caused by *Xanthomonas oryzae* pv. *oryzae* (Xoo) is an economically important disease of rice plants (Yasmin et al., 2017). Heavy reliance on traditional chemicals in its control has resulted in its accumulation, ineffectiveness in plant protection, and environmental unfriendliness. The use of nanoparticles, therefore, alters the common management approach for bacterial diseases by offering a safe alternative for disease suppression and promotion of plant growth (Nair et al., 2010). Due to the many advantages of the use of nanoparticles, they were bio-synthesized and used in our previous studies to break the antibiotic resistance of Xoo *in-vitro* (Abdallah et al., 2019; Ogunyemi et al., 2019). Also, their large surface area and excellent antibacterial properties give them the added advantage of being used as nano-pesticides, which help to improve their release, enhance surface protection, and ultimately reduce agro-environmental pollution (Belkhedkar and Ubale, 2016; Worrall et al., 2018). The ability of nanoparticles to be internalized and bound to bacteria surface due to electrostatic force causing loss of membrane completeness and intracellular contents, ultimately leading to cell death, probably underlies its unique excellent antimicrobial properties (Abdallah et al., 2019).

Magnesium (Mg) is an important element for photo-assimilation and photophosphorylation and is taken by plants as a nanoparticle or ion. Recently, MgONPs were reported by Tamil Elakkiya et al. (2020) to have the ability to increase chlorophyll content by six times. Nano-fertilizers of different metal oxides have the advantage of reducing nutrient losses from plants due to leaching or volatilization, high absorption, and relatively high degradability in comparison to conventional fertilizers (Huang et al., 2018; Salas-Leiva et al., 2021). Manganese (Mn), which is an important micronutrient for plant growth, helps to sustain the metabolic role within plants. It has been reported to be a pivotal cofactor for oxygen-evolving complex (OEC) for photosynthesis and enhancement of water-splitting reaction in photosystem II (PSII; Broadley et al., 2012). In an *in vitro* experiment carried out by Elmer and White (2016), which involved spraying tomato plants with nanoparticles of AlO, ZnO, FeO, CuO, and MnO, wilt disease severity was significantly reduced, and an improvement in the growth of challenged plants was reported.

Green synthesis of materials using various plant parts has been adopted to produce benign nanoparticles. However, the peptides and proteins in micro-organisms in particular bacteriophages make them good reducing, capping, and stabilizing agents in the production of nanoparticles (Lee et al., 2006; Ahiwale et al., 2017), which makes viruses to be used as biological agents in the field of nanotechnology. Recently, M13 bacteriophages have been employed for the preparation of Co_2_O_3_ nanowires and cobalt-platinum crystals (Lee et al., 2006). The 7-11 phage lysates of the family *Podoviridae* have been successfully used to synthesize gold nanoparticles with excellent anti-biofilm activity (Ahiwale et al., 2017). Interestingly, our recent work reported on the Xoo bacteriophage X3, belonging to the *Myoviridae* family (Ogunyemi et al., 2018), which provided a basis upon which to control BLB by employing it to synthesize the new nanomaterials.

Although our recent reports show the antibacterial operation of nanoparticles (Abdallah et al., 2019; Ogunyemi et al., 2019) on the causal organism of BLB, its mechanism is still unclear and phytotoxicity due to ever-increasing exposure of the living organism to them (Capaldi Arruda et al., 2015; Tighe-Neira et al., 2018) is yet to be fully understood. Recently, chlorophyll fluorescence measurements have been used as an informative and useful indicator in characterizing plants to light responses of photosynthesis. The leaf chlorophyll is adopted as an indicator of photosynthetic potential, plant productivity, plant stresses, and senescence (Carter et al., 1996; Carter, 1998). However, there is a dearth of information on the effect of phage-mediated nanoparticles on plants’ photosynthetic ability, chlorophyll synthesis-related genes, and the protection of plants against BLB. Therefore, in this study, we aim to synthesize and characterize MgONPs and MnO_2_NPs using the lysate of phage X3 and evaluate their antibacterial activity against the Xoo strain GZ 0003.

In order to adopt these nanoparticles as safe plant protection agents, the phage-mediated nanoparticles’ phytotoxicity was evaluated by checking their impact on the photosynthesis apparatus of Arabidopsis and, thereafter, their potential use against BLB on rice plants was carried out. Also, to further understand the mechanism of the toxic effect of the nanoparticles on Arabidopsis, we investigated its effect on the chlorophyll synthesis related genes. The results indicated that MgONPs and MnO_2_NPs serve as good antibacterial tools for suppressing bacterial leaf blight disease without having any toxic effect on plants.

## 2. Materials and methods

### 2.1. Preparation of phage lysate and synthesis of MnO_2_NPs and MgONPs

The bacteriophage X3 employed for this study was separated from diseased rice plants in our previous studies (Ogunyemi et al., 2018). MnO_2_NPs and MgONPs were synthesized according to Ahiwale et al. (2017). The phage plaques were re-suspended using SM buffer and then left overnight at 4°C. The plaques in SM buffer were then centrifuged at 10,000 rpm for 10 min at 4°C. Membrane filtering (0.20 μm) was carried out to obtain purified supernatant, which was further applied in the synthesis process by mixing 100 mL of purified phage lysate (1 × 10^10^ PFU/mL) with 100 mL of either 2 mM of MnO_2_ or MgO solution and then incubating the mixtures at room temperature for 74 h. After 10,000 *g* of 20 min centrifugation, the obtained pellets were washed twice with ddH_2_O and freeze-dried using Alpha 1-2 LDplus for further characterization.

### 2.2. Characterization of MnO_2_NPs and MgONPs

The morphology and size of the obtained MnO_2_NPs and MgONPs were determined using transmission electron microscopy (TEM; JEM-1230, JEOL, Akishima, Japan) and scanning electron microscopy (SEM, TM-1000, Hitachi, Japan; Arciniegas-Grijalba et al., 2019), while the elemental composition of the nanoparticles was carried out using SEM mounted with energy dispersive spectrum (EDS; Arciniegas-Grijalba et al., 2019). Functional groups of the obtained MnO_2_NPs and MgONPs were assayed using Fourier transform infrared spectroscopy (FTIR; Vector 22, Bruker, Germany; Ogunyemi et al., 2019) and optical properties of MnO_2_NPs and MgONPs were assayed using UV–Vis spectroscopy (spectrophotometer Cary E 500; Ogunyemi et al., 2019). The crystalline phase present in the synthesized nanoparticles was observed by X-ray diffractometer (XRD), XPert PRO diffractometer (Holland).

### 2.3. *In vitro* antibacterial activity of MnO_2_NPs and MgONPs

Two methods were deployed to investigate the antibacterial impact of 50.0, 100.0, and 200.0 μg/mL MnO_2_NPs and MgONPs on the Xoo strain GZ 0003, which was separated from diseased rice plants of our earlier research (Ogunyemi et al., 2018). The first method was to measure the diameter of inhibition zones on nutrient agar (NA) medium, which was determined by spotting 20 μL of MnO_2_NPs and MgONPs at different concentrations on the bacterized medium, and the plates were kept at 30°C in an incubator for 24 h. The second method was to measure the inhibition of bacterial growth in nutrient broth (NB), which was determined by mixing MnO_2_NPs and MgONPs at different concentrations with an overnight GZ 0003 culture (10^8^ CFU/mL) in a microtiter plate (Corning-Costar Corp., Corning, NY, United States). After incubation at 30°C for 24 h, OD600 nm was measured using a Scanning Microplate Spectrophotometer (Thermo Fisher Scientific Inc., Waltham, MA, United States; Vassallo et al., 2018). Each treatment was repeated three times, and the investigation was also repeated three times.

### 2.4. Effect of MnO_2_NPs and MgONPs on plant chlorophyll

The safety of MnO_2_NPs and MgONPs was evaluated in accordance with Wang et al. (2016) by gauging the chlorophyll fluorescence in Arabidopsis (*Arabidopsis thaliana*) ecotype Columbia (Col-0), which has been adopted as a model plant because of its high sensitivity to light and toxic elements. In brief, germinated seeds of Arabidopsis were sown in pots filled with sterilized soil and subsequently incubated in a growth chamber at 22°C for a light/dark photoperiod of 16/8 h. Four-week old Arabidopsis plants were foliar sprayed with 25 mL of 0, 50, 100, and 200 μg/mL MnO_2_NPs and MgONPs every week followed by subjection to fluorescence signal captured by an in-house fluorescence imaging system (FluorCam, Photon Systems Instruments, Brno, Czech Republic).

Prior to chlorophyll fluorescence measurements, Arabidopsis plants were dark-adapted for approximately 20 min, and a saturating pulse of approximately 1,500 μmol m^−2^ s^−1^ was administered to the plants. This allowed the PSII reaction sites to be closed, and the quantification of the maximum fluorescence yield (*Fm*) in the dark-adapted stage was recorded. Afterward, Arabidopsis leaves were illuminated by an actinic light of 100 μmol m^−2^ s^−1^, which can effectively drive photosynthesis. The highest quantum efficacy of PSII photochemistry in the dark state (*Fv/Fm*) is an important factor in detecting stress levels in plants. The regulated non-photochemical energy dissipation (*ΦNPQ*) is a photo-protective course that eliminates surplus excitation energy, thereby preventing the production of dangerous free radicals. The effective quantum efficacy of PSII photochemistry in the light (*ΦPSII*) that gives a proportion of absorbed light, which is used for photosynthesis, and the coefficient of photochemical quenching (qP), which gives the proportion of reaction centers that are open in photochemistry were measured (Oxborough and Baker, 1997; Kramer et al., 2004; Tietz et al., 2017). The chlorophyll fluorescence technique was adopted to reveal the photosynthetic signature in Arabidopsis plants.

Total RNAs were extracted from 6-week-old *Arabidopsis* leaves using Trizol RNA Kit following the manufacturer’s protocols. Following the treatment with DNase, the integrity and purity of RNAs were evaluated with 1% agarose gel electrophoresis using a Nanodrop spectrometer (Thermo Scientific, Wilmington, United States). The cDNA was synthesized using Goldenstar™ cDNA kit in accordance with the manufacturer company’s instructions, which was subsequently kept at −20°C. Quantitative RT-PCR (qRT-PCR) was employed to determine the effect of MnO_2_NPs and MgONPs on the expression of chlorophylls synthesizing-related genes, including those encoding Magnesium-Protoporphyrin IX Methyltransferase (CHLM), Mg-chelatase subunit D (CHLD), Phytoene Synthase (PSY), Zeta-Carotene Desaturase (ZDS), Chlorophyll A Oxygenase (CAO), Chlorophyll Synthase (CHLG), Copper Response Defect (CRD), Photosystem I Subunit D-2 (PSAD-2), and Photosystem I Subunit E-2 (PSAE-2). Arabidopsis gene *ACTIN2* (*ACT2*) was employed as an internal check. The primers used have been described previously (Qin et al., 2007; Stephenson and Terry, 2008; Wang et al., 2016), as shown in Table 1.

**Table 1:** Primers used in this study.

Primers	Sequence	References
CHLG-F	5′-GAGATTTGTTGTGCGTGCGG-3′	Wang et al. (2016)
CHLG-R	5′-CCAGTGGAGGCCAAGTGACT-3′	Wang et al. (2016)
CAO-F	5′-AGTCCTTCTGCTTTATCTCTC-3′	Wang et al. (2016)
CAO-R	5′-TTCTCAACTAATCCACTCTCA-3′	Wang et al. (2016)
CRD-F	5′-AAGAGGAAACTGGATAGAA-3′	Wang et al. (2016)
CRD-R	5′-AAAGAAGTAACCAAAGGAA-3′	Wang et al. (2016)
CHLM-F	5′-AGCCGGGGTCGACAGTACAACAATC-3′	Stephenson and Terry (2008)
CHLM-R	5′-ACCGGCCAAGGATCTATCTTCAGTC-3′	Stephenson and Terry (2008)
CHLD-F	5′-CCACATCAGATACGGATACGG-3’	Stephenson and Terry (2008)
CHLD-R	5′-GTCAGCATTGTACTCTATGCGCTC-3′	Stephenson and Terry (2008)
PSAD-2-F	5′-CAAACACACCATCACCAATC-3′	Wang et al. (2016)
PSAD-2-R	5′-ACCTCGTACCTAAAGCCAAA-3′	Wang et al. (2016)
PSAE-2-F	5′-CACCACCATTGTGTCTTTCT-3′	Wang et al. (2016)
PSAE-2-R	5′-TTGACCTTGGATCCTCTCTT-3′	Wang et al. (2016)
ZDS-F	5′-AGATAGAGGTGGCAGAATCC-3′	Qin et al. (2007)
ZDS-R	5′-GGTGTTAGAACGCACTGAAG-3′	Qin et al. (2007)
PSY-F	5′-GAACCGAAGTAGAAGAATTG-3′	Qin et al. (2007)
PSY-R	5′-GATCATCGAAGTTCTGGT-3′	Qin et al. (2007)

### 2.5. *In vivo* antibacterial effect of MnO_2_NPs and MgONPs

The role of MnO_2_NPs and MgONPs in protecting rice plants from BLB infection was assessed according to the method described by Yasmin et al. (2017). In brief, germinated rice seeds (cv. II You 023 *Oryza sativa* L.) were planted in small pots filled with sterile soil and kept in the growth chamber at 28°C ± 2°C, at 80% relative humidity, with a photoperiod of 16 light hours and 8 dark hours. The pots were arranged in a completely randomized block design (CRD) maintaining three repeats for individual treatment. At the fourth-leaf growth stage, rice plants were inoculated with GZ 0003 cell suspension (10^8^ CFU/mL) via leaf clipping and were applied with 50 mL of 200.0 μg/mL of MnO_2_NPs and MgONPs by foliar spray 24 h post-inoculation with the bacterial pathogen. Lesion length was measured 14 days post-inoculation with the bacterial pathogen, and the diseased leaf area (DLA) was determined based on the proportion of lesion leaves according to Yasmin et al. (2017). Double distilled water and bacterial pathogen alone served as negative and positive controls.

### 2.6. Antibacterial mechanism of MnO_2_NPs and MgONPs

The antibacterial mechanism of MnO_2_NPs and MgONPs was determined in this study based on their effect on biofilm formation, the outflow of intracellular materials, cell morphology, and the apoptosis of strain GZ 0003. Biofilm development was carried out using a 96-well plate according to the crystal violet staining method (Hassan et al., 2011). Briefly, individual plates were inoculated with a 100 μL GZ 0003 culture (10^8^ CFU/mL) amended with 20 μL of 0.0, 50.0, 100.0, and 200.0 μg/mL MnO_2_NPs and MgONPs. After having been kept static at 30°C in an incubator for 24 h to ensure bacterial biofilm adhesion, OD_570_ was quantified using a Scanning Microplate Spectrophotometer (Thermo Fisher Scientific Inc., Waltham, MA, United States). The outflow of intracellular materials in strain GZ 0003 was monitored using UV–Vis spectrophotometry at 260 nm according to Chen et al. (2013). GZ 0003 was treated using 0.0, 50.0, 100.0, and 200.0 μg/mL of either MnO_2_NPs or MgONPs and was kept at 30°C in an incubator for 4 h. The impact of MnO_2_NPs and MgONPs on cell morphology was observed as described by Helander et al. (2001) under TEM (JEM-1230, JEOL, Tokyo, Japan). Bacterial culture (1 mL) was treated with MnO_2_NPs and MgONPs (200.0 μg/mL) and kept at 30°C in an incubator for 20 min. Apoptosis was detected using propidium iodide in accordance with the protocol of Cai et al. (2018), i.e., by observing the changes in the light scattering under a flow cytometer (Beckman Coulter, Gallios, Germany; Cai et al., 2018). Bacterial cells were treated with 0.0 or 200.0 μg/mL of MnO_2_NPs and MgONPs for 4 h. Individual treatment had three repeats, and the investigation was carried out three times.

### 2.7. Statistical analysis

Statistical analysis of the obtained results from the present study was carried out using SAS software (version 9.1.3, 2003) and the data were analyzed via a two-way factorial design. The results were expressed as mean ± SEM. The significance of differences between means was calculated using the Duncan’s test (Duncan, 1955). A statistically significant difference was set at *p* < 0.05.

## 3. Results

### 3.1. Biological synthesis and characterization of MgONPs and MnO_2_NPs

**Figure 1:**
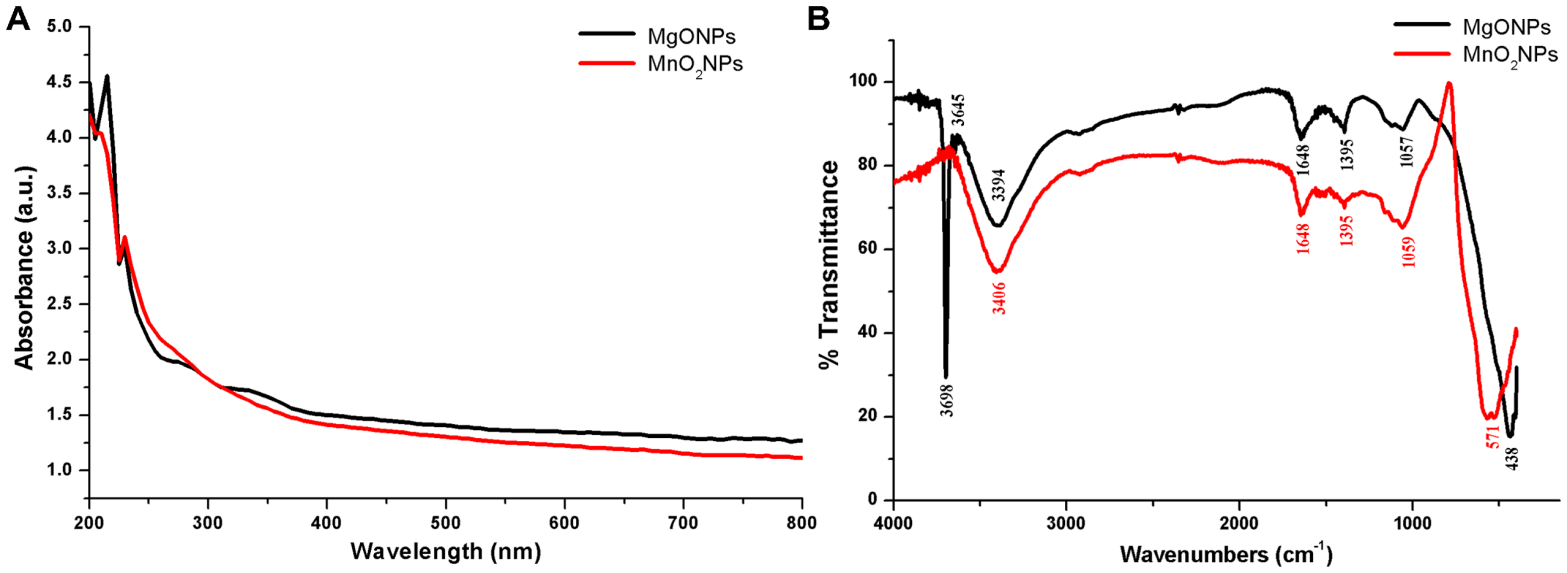
Characterization of the bio-synthesized MgONPs and MnO_2_NPs. **(A)** Analysis of UV—Vis spectra. **(B)** Fourier transform infrared spectra.

UV–Vis spectrophotometer was carried out for the validation of the formation of MgONPs and MnO_2_NPs synthesized by phage lysate X3 (Figure 1A). As shown in Figure 1A, MgONPs had an absorption peak at 215 nm, whereas the MnO_2_NPs peak was observed at 230 nm after 74 h of incubation time (Figure 1A). Spectra of MgONPs and MnO_2_NPs using phage lysate X3 are presented in Figure 1B. The bands were shown at 3,698, 3,645, 3,394, 1,648, 1,395, 1,057, and 438 cm^−1^ for MgONPs, while the IR spectrum MnO_2_NPs bands were at 3,406, 1,648, 1,395, 1,059, and 571 cm^−1^ (Figure 1B).

For the MgONPs, the very strong, broad, and sharp absorptance peaks at 3,698 and 3,645 cm^−1^ equate to strong stretching vibrations of (O–H); 3,394 cm^−1^ correlates to strong, broad stretching of the H bonded vibration of the O–H group and the N–H stretching of the primary amine group. The C=C stretching vibration and strong C=O stretching vibration are assigned to the band at 1,648 cm^−1^, while C–H bending vibration is ascribed to the band at 1,395 cm^−1^. The band in 1,057 cm^−1^ corresponds to C–O stretching vibration, while 438 cm^−1^ correlates to Mg–O stretching vibration (Figure 1B). The strong broadband at 3,406 cm^−1^ of the MnO_2_NPs spectrum corresponds to the O–H stretching vibration and N–H stretching of the primary amine group, 1,648 cm^−1^ is assigned to C=C stretching vibration and a strong C=O stretching vibration. C–H bending vibration is ascribed to the band at 1,395 cm^−1^, while C–O stretching vibration is assigned to the band at 1,059 cm^−1^. Band 571 cm^−1^ is a typical band for Mn–O stretching vibration of the tetrahedron and octahedron sites, as depicted in Figure 1B.

**Figure 2:**
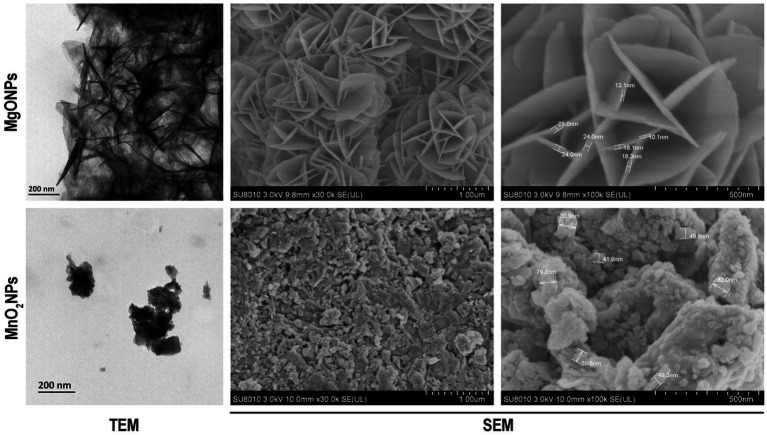
Characterization of the bio-synthesized MgONPs and MnO_2_NPs by the assay of transmission electron micrographs; and scanning electron micrographs.

TEM and SEM of MgONPs and MnO_2_NPs were carried out to reveal the particle structure and size of the phage lysate synthesized nanoparticles (Figure 2). The sample revealed an irregularly spherical shape for MnO_2_NPs, while a flower shape (nanoflower) was observed for MgONPs. The particle size range for MnO_2_NPs was 41.9 to 82.0 nm, while MgONPs had a size range of 10.1 to 25.0 nm (Figure 2).

**Figure 3:**
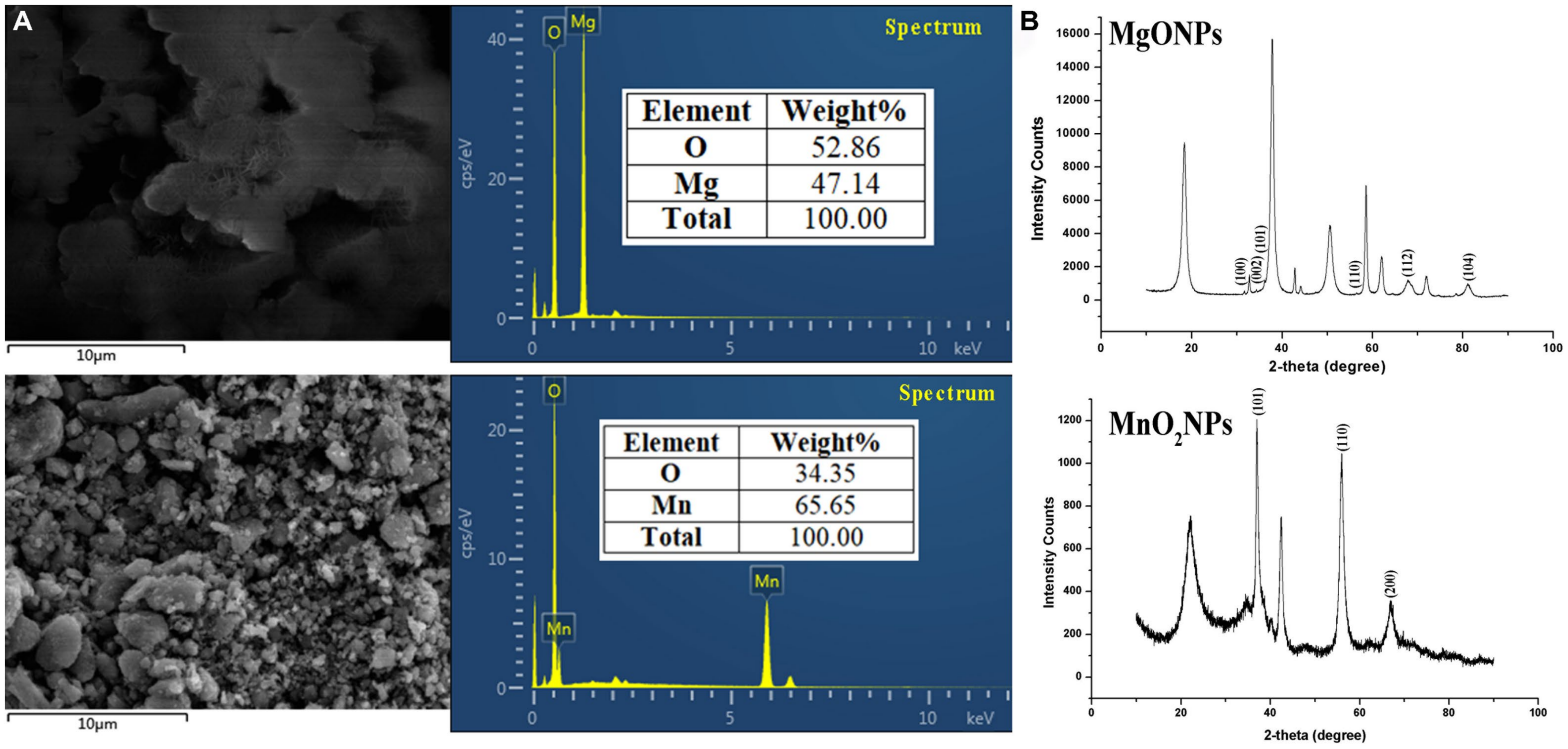
Characterization of bio-synthesized MgONPs and MnO_2_NPs. **(A)** Analysis of energy dispersive spectrum; **(B)** Analysis of X-ray diffractometer spectra.

The presence of Mn and Mg elements in MnO_2_NPs and MgONPs, respectively, was ascertained using an EDS instrument. The percentage elemental composition was 47.14 Mg and 65.65 Mn (Figure 3A). The strong intensity peaks at 1.5 and 6.0 keV for magnesium and manganese, respectively, indicate the reduction of Mg ion and Mn ion to their 0 valence state (Figure 3A). XRD results of the MgONPs and MnO_2_NPs powder are presented in Figure 3B. Three characteristic peaks were recognized for MnO_2_NPs. Diffraction characteristics are displayed in 2Ɵ degrees for MnO_2_NPs as 37.04, 56.00, and 67.01, corresponding to crystal planes of (101), (110), and (200), whereas 2Ɵ degrees of MgONPs are 31.72, 34.37, 36.20, 56.56, 67.85, and 81.14, corresponding to crystal planes (100), (002), (101), (110), (112), and (104; Figure 3B). The mean particle sizes, in accordance with Scherrer’s formula, were found to be 9.8 and 12.5 nm for MnO_2_NPs and MgONPs, respectively.

### 3.2. *In vitro* antibacterial operation of MgONPs and MnO_2_NPs

**Figure 4:**
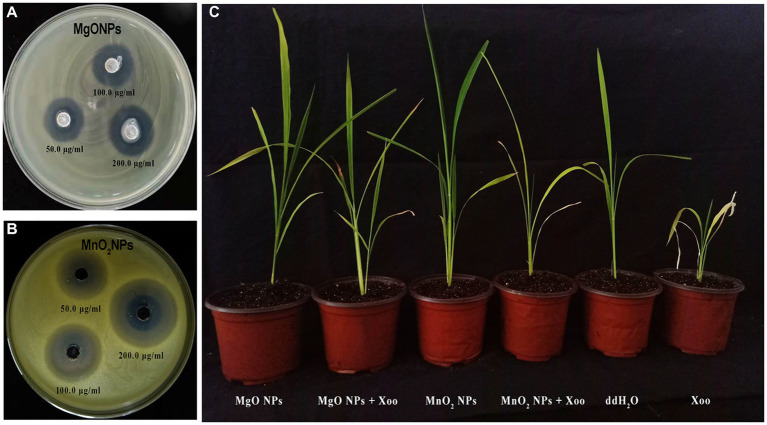
Pictorial representation of: **(A,B)** Antagonistic activity of the bio-synthesized MgONPs and MnO_2_NPs on GZ 0003 NA plate; **(C)** Effect of MgONPs and MnO_2_NPs on rice plant.

Herein, the antagonist reaction of MgONPs and MnO_2_NPs at various concentrations (50, 100, and 200 μg/mL) against GZ 0003 in the form of clearing zones were 3.5 and 2.1 cm, respectively, for MnO_2_NPs and MgONPs at 200 μg/mL after having been kept for 24 h in an incubator (Figure 4; Table 2). The bacterial number decreased by 77.78% and 71.47%, respectively, for MgONPs and MnO_2_NPs at 200 μg/mL (Table 2). The maximum inhibition of growth of GZ 0003 was 200 μg/mL at OD600 (Table 2). A two-way ANOVA analysis was used to reveal the effect of nanoparticles and concentration on the antibacterial operation of bio-synthesized MgONPs and MnO_2_NPs on GZ 0003. MnO_2_NPs had the most significant effect on antagonism at 200 μg/mL, while MgONPs at 200 μg/mL had the most significant reduction in bacterial growth (Table 2).

**Table 2:** Antibacterial operation of bio-synthesized MgONPs and MnO_2_NPs on GZ 0003.

Parameters classification	Bacterial growth in NB (OD 600 nm)	Biofilm formation (OD 570 nm)	Antagonistic activity in NA (cm)	Cell efflux (OD 260 nm)
**(A) Effect of nanoparticles**				
Control	1.261^a^	1.554^a^	0.000^c^	0.042^c^
MgONPs	0.575^b^	0.663^b^	1.711^b^	0.305^a^
MnO_2_NPs	0.541^b^	0.827^b^	2.988^a^	0.231^b^
^*^SEM	0.018	0.053	0.067	0.004
Significance	0.0001	0.0001	0.042	0.0001
**(B) Effect of concentration of nanoparticles (μg/mL)**				
0.0	1.261^a^	1.554^a^	0.000^d^	0.042^b^
50.0	0.710^b^	0.822^b^	1.500^c^	0.232^a^
100.0	0.551^bc^	0.740^b^	2.366^b^	0.292^a^
200.0	0.414^b^	0.673^b^	3.183^a^	0.280^a^
^*^SEM	0.018	0.053	0.067	0.004
Significance	0.0001	0.003	0.001	0.0001
(A*B) Effect of interaction between nanoparticles and concentrations				
Control	1.261^a^	1.554^a^	0.000^g^	0.042^e^
MgONPs 50 (μg/mL)	0.869^b^	1.098^b^	1.267^f^	0.161^cd^
MgONPs 100 (μg/mL)	0.551^c^	0.546^d^	1.733^e^	0.303^b^
MgONPs 200 (μg/mL)	0.306^d^	0.345^e^	2.133^d^	0.451^a^
MnO_2_NPs 50 (μg/mL)	0.796^b^	1.135^b^	2.600^c^	0.134^d^
MnO_2_NPs 100 (μg/mL)	0.535^c^	0.903^c^	2.867^b^	0.231^c^
MnO_2_NPs 200 (μg/mL)	0.294^d^	0.444^de^	3.500^a^	0.330^b^
^*^SEM	0.037	0.044	0.059	0.023
Significance	0.0001	0.0001	0.0001	0.0005

Values with the same letters in each column are not significantly different at (*P* < 0.05). NA, Nutrient agar; NB, Nutrient broth. ^*^SEM, Standard Error of the Mean; NS, Not significant.

### 3.3. MgONPs and MnO_2_NPs are safe to plant by increasing chlorophyll

Chlorophyll fluorescence in response to different concentrations of MgONPs and MnO_2_NPs treatment is shown in Table 3. The *Fv/Fm* was approximately 0.80 for both the control and the treatment, which were not significantly different, reflecting that the plants were not subjected to any toxic effects as a result of the treatment (Table 3). According to two-way ANOVA analysis, the ΦPSII was significantly increased in the treatment with MgONPs and MnO_2_NPs, respectively, in comparison to the control, whereas no significant difference was recorded in qP when treated with MgONPs and MnO_2_NPs in comparison to the control (Table 3). For the MgONPs treatment, ΦNPQ was significantly reduced by 39.11%, 62.38%, and 50.00% at 50, 100, and 200 μg/mL, respectively (Table 3). Reductions of 25.25%, 57.43%, and 50.99% at 50, 100, and 200 μg/mL, respectively, were observed when the plants were treated with MnO_2_NPs for ΦNPQ (Table 3).

**Table 3:** Effect of MgONPs and MnO_2_NPs on chlorophyll fluorescence emission of *Arabidopsis thaliana* ecotype Columbia (Col-0).

Parameters classification	Fv/Fm	ΦPSII	ΦNPQ	QP
**(A) Effect of nanoparticles**				
Control	0.820^a^	0.299^b^	2.020^a^	0.660^b^
MgONPs	0.806^ab^	0.593^a^	1.000^b^	0.920^a^
MnO_2_NPs	0.783^b^	0.586^a^	1.119^b^	0.928^a^
^*^SEM	0.005	0.004	0.045	0.009
Significance	0.014	0.050	0.0006	0.002
**(B) Effect of concentration of nanoparticles (μg/mL)**				
0.0	0.82	0.299^b^	2.020^a^	0.660^b^
50.0	0.799	0.585^a^	1.119^b^	0.921^a^
100.0	0.795	0.555^a^	1.134^b^	0.923^a^
200.0	0.789	0.630^a^	0.925^b^	0.929^a^
^*^SEM	0.005	0.004	0.045	0.009
Significance	0.232^NS^	0.0001	0.001	0.0053
(A*B) Effect of interaction between nanoparticles and concentrations				
Control	0.82	0.299^c^	2.020^a^	0.66
MgONPs 50 (μg/mL)	0.8	0.561^ab^	1.230^bc^	0.871
MgONPs 100 (μg/mL)	0.8	0.610^ab^	1.009^cd^	0.971
MgONPs 200 (μg/mL)	0.82	0.610^ab^	0.762^d^	0.92
MnO_2_NPs 50 (μg/mL)	0.77	0.500^b^	1.508^b^	0.927
MnO_2_NPs 100 (μg/mL)	0.79	0.610^ab^	0.860^cd^	0.948
MnO_2_NPs 200 (μg/mL)	0.79	0.650^a^	0.990^cd^	0.91
^*^SEM	0.013	0.038	0.122	0.056
Significance	0.999^NS^	0.0002	0.001	0.437^NS^

Values with the same letters in each column are not significantly different at (*P* < 0.05). Fv/Fm, Quantum efficiency of Photosystem II; ΦPSII, Effective quantum efficiency of PSII photochemistry in the light; ΦNPQ, Non-photochemical energy dissipation; QP, Photochemical quenching; ^*^SEM, Standard Error of the Mean; NS, Not significant.

Notably, A260/A280 values between 1.8 and 2.2, which indicated a good quality sample of the total RNA was used as a template for cDNA synthesis. Expression levels of chlorophyll synthesis (CHLG, CAO, CRD, CHLM, and CHLD), carotenoid synthesis (ZDS and PSY), and photosystem structure genes (PSAD-2 and PSAE-2), which are all integral parts of plant photosynthesis are shown in Tables 4, 5. Expression levels of the chlorophyll synthesis and photosystem structure genes tested increased at all concentrations in MgONPs and MnO_2_NPs application in comparison to the control (Tables 4, 5). Furthermore, two-way ANOVA analysis detected that 200 μg/mL had the most significant effect on chlorophyll synthesis genes, while MgONPs at 200 μg/mL had the most significant effect on photosystem structure genes compared to the other interactions. Conversely, expression levels of carotenoid synthesis genes tested decreased at all concentrations for the MgONPs and MnO_2_NPs treatment compared to the control (Table 5).

**Table 4:** Effect of the bio-synthesized MgONPs and MnO2NPs on the expression of chlorophyll synthesis genes in *Arabidopsis thaliana* ecotype Columbia (Col-0).

Parameters classification	CHLG	CAO	CRD	CHLM	CHLD
**(A) Effect of nanoparticles**					
Control	1.001^b^	1.006^b^	1.050^c^	1.098^b^	1.066^b^
MgONPs	1.883^a^	1.853^a^	2.145^a^	2.386^b^	3.020^a^
MnO_2_NPs	1.936^a^	2.009^a^	2.326^a^	2.587^a^	2.347^a^
^*^SEM	0.125	0.01	0.14	0.177	0.756
Significance	0.001	0.001	0.012	0.012	0.007
**(B) Effect of concentration of nanoparticles (μg/mL)**					
0.0	1.001^c^	1.006^c^	1.050^c^	1.098^c^	1.065^b^
50.0	1.622^b^	1.596^b^	1.847^b^	2.055^b^	2.560^a^
100.0	1.869^ab^	1.992^ab^	2.306^ab^	2.564^ab^	2.861^a^
200.0	2.238^a^	2.205^a^	2.553^a^	2.840^a^	2.631^a^
^*^SEM	0.125	0.01	0.14	0.177	0.756
Significance	0.0008	0.002	0.002	0.002	0.050
(A*B) Effect of interaction between nanoparticles and concentrations					
Control	1.001^d^	1.006^c^	1.050^c^	1.098^c^	1.066^c^
MgONPs 50 (μg/mL)	1.516^bc^	1.492^b^	1.727^b^	1.921^b^	2.140^bc^
MgONPs 100 (μg/mL)	1.728^bc^	1.700^b^	1.969^b^	2.190^b^	2.980^ab^
MgONPs 200 (μg/mL)	2.405^a^	2.367^a^	2.740^a^	3.048^a^	3.941^a^
MnO_2_NPs 50 (μg/mL)	1.333^cd^	1.617^b^	1.872^b^	2.081^b^	1.781^bc^
MnO_2_NPs 100 (μg/mL)	1.874^b^	1.844^b^	2.135^b^	2.375^b^	2.462^ab^
MnO_2_NPs 200 (μg/mL)	2.603^a^	2.567^a^	2.971^a^	3.305^a^	2.800^ab^
^*^SEM	0.165	0.140	0.163	0.184	0.508
Significance	0.019	0.007	0.007	0.008	0.031

Values with the same letters in each column are not significantly different at (*P* < 0.05). CHLG, Chlorophyll Synthase; CAO, Chlorophyll A Oxygenase; CRD, Copper Response Defect; CHLM, Magnesium-Protoporphyrin IX Methyltransferase; CHLD, Mg-chelatase subunit D; ^*^SEM, Standard Error of the Mean; NS, Not significant.

**Table 5:** Effect of MgONPs and MnO_2_NPs on the expression of photosystem structure genes and carotenoid synthesis genes in *Arabidopsis thaliana* ecotype Columbia (Col-0).

Parameters classification	PSAD-2	PSAE-2	ZDS	PSY
**(A) Effect of nanoparticles**				
Control	1.091^b^	1.063^b^	1.052^a^	1.039^a^
MgONPs	2.489^a^	2.324^b^	0.585^b^	0.610^b^
MnO_2_NPs	2.183^a^	1.824^ab^	0.635^b^	0.627^b^
^*^SEM	0.362	0.397	0.045	0.042
Significance	0.003	0.012	0.018	0.045
**(B) Effect of concentration of nanoparticles (μg/mL)**				
0.0	1.091^b^	1.063^b^	1.052^a^	1.039^a^
50.0	2.107^a^	2.003^a^	0.679^b^	0.700^b^
100.0	2.425^a^	2.242^a^	0.625^b^	0.611^b^
200.0	2.477^a^	1.978^a^	0.526^b^	0.545^b^
^*^SEM	0.362	0.397	0.045	0.009
Significance	0.013	0.086^NS^	0.036	0.005
(A*B) Effect of interaction between nanoparticles and concentrations				
Control	1.091^d^	1.063^c^	1.052^a^	1.039^a^
MgONPs 50 (μg/mL)	1.677^cd^	1.609^bc^	0.787^abc^	0.800^ab^
MgONPs 100 (μg/mL)	2.537^abc^	2.398^ab^	0.573^bc^	0.601^b^
MgONPs 200 (μg/mL)	3.254^a^	2.967^a^	0.397^c^	0.430^b^
MnO_2_NPs 50 (μg/mL)	1.596^cd^	1.517^bc^	0.853^ab^	0.793^ab^
MnO_2_NPs 100 (μg/mL)	2.160^bc^	1.666^bc^	0.621^bc^	0.651^b^
MnO_2_NPs 200 (μg/mL)	2.795^ab^	2.290^ab^	0.431^c^	0.439^b^
^*^SEM	0.312	0.339	0.119	0.114
Significance	0.003	0.021	0.001	0.022

Values with the same letters are not significantly different at (*P* < 0.05). PSAD-2, Photosystem I Subunit D-2; PSAE-2, Photosystem I Subunit E-2; ZDS, Zeta-Carotene Desaturase; PSY, Phytoene Synthase; ^*^SEM, Standard Error of the Mean; NS, Not significant.

### 3.4. MgONPs and MnO_2_NPs could protect plants from bacterial infection

**Figure 5:**
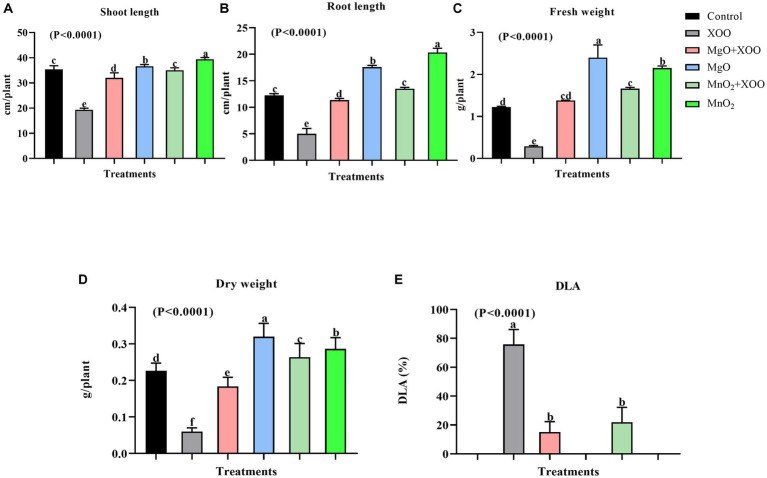
Effect of the bio-synthesized MgONPs and MnO_2_NPs on rice plant growth parameters. **(A)** Shoot Length; **(B)** Root Length; **(C)** Fresh Weight; **(D)** Dry Weight; **(E)** Percentage diseased leaf area (DLA %). *Values are mean ± standard deviation of three repeats and bars with the same letters are not significantly different at (*p* < 0.05).

Since the nanoparticles were found to have no toxic effect on Arabidopsis and significantly improved the chlorophyll fluorescence parameters, their efficiency in suppressing rice BLB was studied in a pot experiment (Figures 4, 5). The foliar spray of 200 μg/mL of MgONPs and MnO_2_NPs resulted in significant disease suppression. Diseased leaf areas of 15.04% and 21.86% for MgONPs and MnO_2_NPs treatment, respectively, were observed against the control DLA of 75.79% (Figure 5E).

As shown in Figures 4, 5, plants treated with MgONPs and MnO_2_NPs only had an increment in shoot length and root length and a significant increase in fresh and dry weight in comparison to other treatments. The application of MgONPs and MnO_2_NPs on Xoo-infected plants significantly increased the shoot length, root length, and fresh and dry weight compared to untreated Xoo-infected plants (Figures 5A–D). Notably, MnO_2_NPs significantly increased the shoot length and root length, which was statistically different from the other treatments. Furthermore, the treatment of the Xoo-infected plant with MnO_2_NPs significantly increased its shoot length and root length compared to treatment with MgONPs (Figures 5A,B).

### 3.5. Various mechanisms involved in the antibacterial activity of MgONPs and MnO_2_NPs

**Figure 6:**
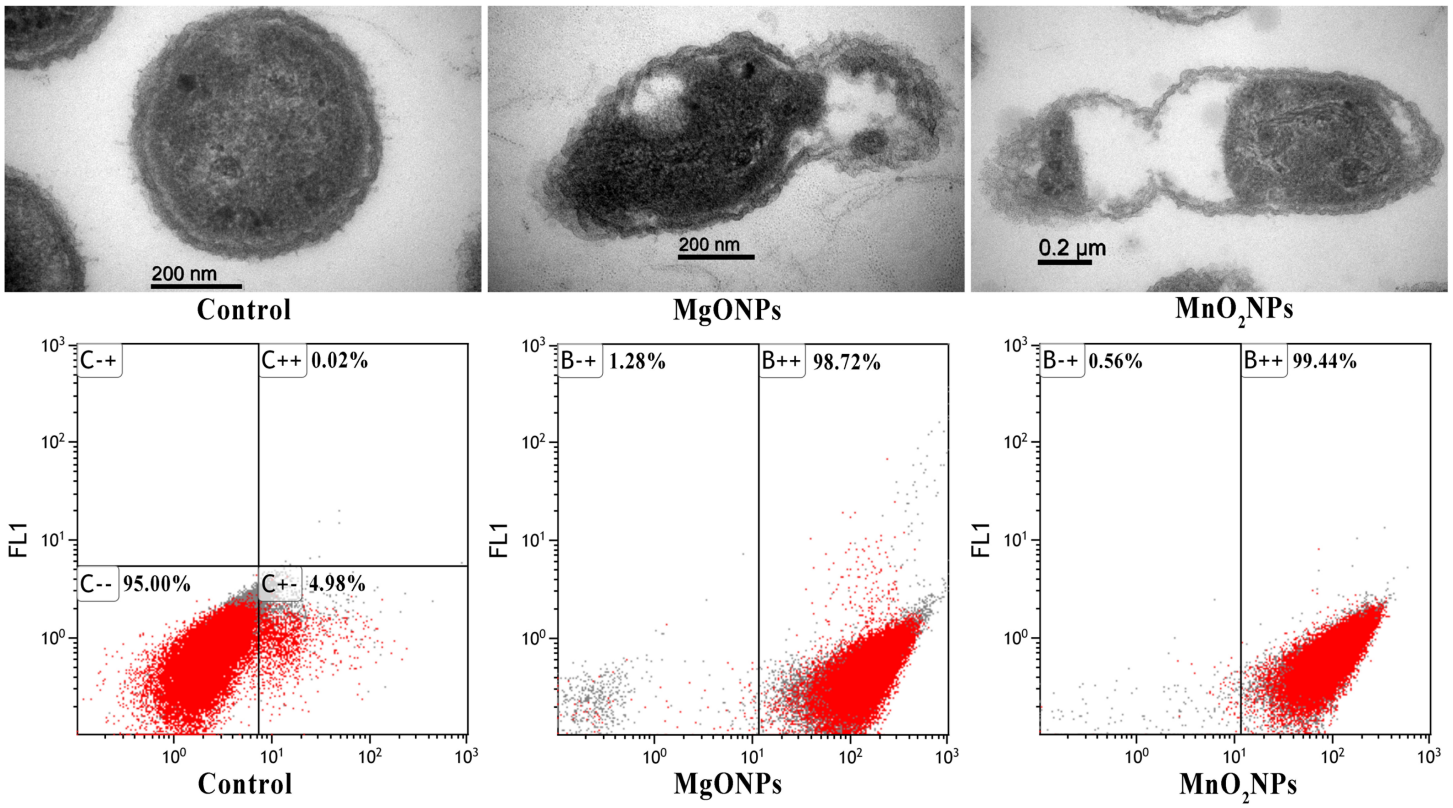
TEM images of GZ 0003 treated with ddH_2_O (control); and 200.0 μg/mL of the bio-synthesized MgONPs and MnO_2_NPs; and Flow cytometry images of GZ 0003 after incubation with ddH_2_O (control) and bio-synthesized MgONPs and MnO2NPs.

The efficacy of the nanoparticles as good control agents was studied according to their antibiofilm effect on the Xoo strain GZ 0003, the causative organism of BLB (Table 2). The nanoparticles at all concentrations (50, 100, and 200 μg/mL) caused a significant biofilm, with reductions of 75.77% and 76.72% at 200 μg/mL for MgONPs and MnO_2_NPs, respectively (Table 2). However, using two-way ANOVA analysis, it was revealed that MgONPs and MnO_2_NPs at 200 μg/mL were not significantly different from each other (Table 2). Considering the antibacterial activity of MgONPs and MnO_2_NPs, it was mandatory to evaluate its mechanism of operation. The effects of 200 μg/mL MgONPs and MnO_2_NPs on the morphology of Xoo cells were compared to the control (Figure 6). Structural distortion of GZ 0003 cell membranes and the loss of cellular content were noted in treated cells, while the untreated cells maintained typical Xoo cell morphology, with full cellular content (Figure 6). Apoptosis was found to have increased from 5% when treated with double distilled water to 98.72% and 99.44% for 200 μg/mL MgONPs and MnO_2_NPs treatment, respectively (Figure 6).

As a result of the empty segment of the cell observed, the efflux of cellular material was determined. Significant effluxes of cellular material of 0.45 and 0.33 at A260 for 200.0 μg/mL of MgONPs and MnO_2_NPs, respectively, were found (Table 2). Using two-way ANOVA analysis, MgONPs at 200.0 μg/mL were observed to have an increased cellular efflux, which was statistically significantly different from MnO_2_NPs (Table 2).

## 4. Discussion

The absorbance peaks observed confirm the formation of nanoparticles. Similar absorbance peaks were reported for *Penicillium chrysogenum* and unpasteurized cow milk bio-synthesized MgONPs (Mirhosseini and Afzali, 2016; El-Sayyad et al., 2018). The production of nanoparticles using viruses is a safe approach for bio-synthesis reaction because of their non-toxic properties, which provides a natural reduction and capping route for the nanoparticles (Lee et al., 2006; Ahiwale et al., 2017). It has been hypothesized that the interaction of biomolecules, particularly peptides, on the metal surface leads to the stability of nanoparticles and improves their use as sensors, biomedical devices, and electronics. In order to develop peptides that can precisely bind to the surface of metal materials, the peptide-substrate interaction connection was shown via the phage display method (Sharma et al., 2019). Also, in our previous work, it was reported that only the protein component of *Paenibacillus polymyxa* strain Sx3 was responsible for the synthesis of MgO, ZnO, and MnO_2_ nanoparticles when compared to the control (Ogunyemi et al., 2020). Hence, the nanoparticles produced using phage X3 lysate in this study were from its protein component and were devoid of any toxic components.

Investigation of the IR spectrum was performed to obtain information about functional groups present in the synthesized MgONPs and MnO_2_NPs. The FTIR helped to identify the interaction between MgO, MnO_2,_ and bioactive constituents, which is liable for the synthesis and stabilization of MgONPs and MnO_2_NPs. A peak of 438 cm^−1^ corresponds to Mg–O stretching vibration (Prescott et al., 2005), while the band noted at 571 cm^−1^ is representative of the typical band for Mn–O stretching vibration of the tetrahedron and octahedron centers (Ozkaya et al., 2008; Krishnan and Mahalingam, 2017). The MgONPs strong, sharp band at 3698 cm^−1^ is linked to individual coordinates of hydroxides present in the nanopowder serving as a proton acceptor (Kwon and Park, 2009; Chanda et al., 2017). The strong interaction of water molecules with the MgO and MnO_2_ top layer might be responsible for the O–H group. The N–H, C=C, C–H, C=O, and C–O functional groups common to phage X3 synthesized MgONPs and MnO_2_NPs are due to the presence of phage protein synthesized during nanoparticle formation (Irais Vera-Robles et al., 2016; Fouad et al., 2017). Previous studies have reported phage absorption peaks at 3,300, 1,631, 1,529, 1,390, and 1,053 cm^−1^, which imply the O–H and N–H stretching, N–H bending, C–N stretching, C–H bending, and O–C=O stretching, respectively (Dong et al., 2013; Lai et al., 2021). The amino acids functional groups reported to be contained in the phage reduced the metal ions during the synthesis of nanoparticles. The functional group O–H, C=O, C–H, C–N, N–H, and C=C contained in all the nanoparticles is the amino acid residues and protein synthesized. Therefore, based on the functional groups observed in MgONPs and MnO_2_NPs, we conclude that the protein in the bacteriophage X3 had a pivotal role in the reduction of the nanoparticles.

Proteins have been reported to be significant components in bio-mediating the synthesis process of nanoparticles, which binds it by amine groups or cysteine remnants in proteins via electrostatic pull (Naik et al., 2002; Sanghi and Verma, 2009). The percentage elemental composition of 47.14 Mg and 65.65 Mn noted in this examination is in agreement with earlier studies (Madzokere and Karthigeyan, 2017; Farzana et al., 2018). The TEM and SEM are techniques employed to know the structural morphology of the NPs while giving a particle size range. However, the XRD technique uses the highest peak to determine the dominant size of the nanoparticles according to Scherrer’s formula (Bokuniaeva and Vorokh, 2019). Hence, according to Scherrer’s formula, the mean particle sizes of the nanoparticles were concluded to be 9.8 and 12.5 nm for MnO_2_NPs and MgONPs, respectively.

Recently, the excellent antimicrobial activity of micro-organism bio-inspired MgONPs and MnO_2_NPs on different microbial pathogens were reported (El-Sayyad et al., 2018; Arasu et al., 2019; Saka et al., 2022). Likewise, the bacterial number of strain GZ 0003 was significantly reduced at all concentrations (50, 100, and 200 μg/mL) when treated with MgONPs and MnO_2_NPs. Furthermore, from the two-way ANOVA analysis, it was discovered that MnO_2_NPs at 200 μg/mL had the greatest antagonist effect in the NA media plate in comparison to other interactions. Moreover, for bacterial growth, MgONPs at 200 μg/mL had the most significant reduction in bacterial number compared to other interactions. According to numerous studies, metal oxide NPs have drawn a lot of attention because of their antibacterial action, which is based on their small size and allows them to penetrate bacteria and damage internal components (Joshi et al., 2020). There is insufficient information on the antibacterial operation of MnO_2_NPs; most studies focus on its application in electronic properties and catalytic activities (Hoseinpour and Ghaemi, 2018); therefore, the antibacterial activity reports in this study help to bridge the gap.

The chlorophyll fluorescence experiment was carried out on Arabidopsis because of its high sensitivity to light and toxicity. The conserved ratio of *Fv/Fm* for the healthy plant is approximately 0.80–0.85, while a decrease in this value is indicative of stress to the plants (Baker, 2008). From the two-way ANOVA analysis, it was shown that there was no significant difference between MgONPs and MnO_2_NPs at all concentrations when compared with the control. The *Fv/Fm* ratio was approximately between 0.80 and 0.82. It has been reported that the photosynthetic efficacy of plants reduces when they are exposed to stress conditions such as toxicity (Zhao et al., 2007; Santos et al., 2023; Vitale et al., 2023). Hence, the result of the chlorophyll fluorescence revealed that treatment with MgONPs and MnO_2_NPs positively enhanced the chlorophyll fluorescence parameters of Arabidopsis without toxic effects on the plants. Over the years, chlorophyll fluorescence has been employed as a benchmark to examine the content of chlorophyll in plants and detect plant stresses caused by exposure to hazardous materials (Ndao et al., 2005; Peroti et al., 2021). This study, therefore, shows that the application of the phage-mediated nanoparticles on Arabidopsis had no significant toxic effect on plants.

The result of expression levels of chlorophyll synthesis, carotenoid synthesis, and photosystem structure genes of this research contradicts the reports of Wang et al. (2016), who reported a decrease in the chlorophyll synthesis related genes of Arabidopsis due to zinc oxide nanoparticle treatment. The mechanism of chlorophyll improvement for metal oxide nanoparticles has been reported to be involved in its ability to penetrate the plant chloroplast and reach the photo system-II (PS-II) reaction center, thereby increasing the transmission of electrons and light absorption in chloroplasts. The small size of nanoparticles gives them the advantage of being able to navigate in the chlorophyllase (chlorophyll synthesis enzymes), which in turn improves photosynthetic efficiency. Furthermore, NPs enhance plant photosynthetic efficiency by promoting the electron proton transportation chain. Ribulose-1,5-bisphosphate carboxylase/oxygenase (RuBisCO) activity, nitrogen assimilation, and nitrate reductase activity have been documented to be improved, which invariably improves photosynthetic efficiency in plants. NPs also improve light and O_2_ absorption in the chloroplast, which consequently improves plant photosynthetic efficiency. NP application in plants enhances the uptake of Calcium, Magnesium, Nitrogen, Potassium, and iron while improving gas exchange characteristics, which ensures a better photosynthesis process (Faraji and Sepehri, 2020; Ali et al., 2021; Rasheed et al., 2022). The quantity of chlorophyll in a plant is directly related to its photosynthetic ability, production, and yield potential (Xue and Yang, 2009). According to two-way ANOVA analysis, MgONPs and MnO_2_NPs at 200 μg/mL had the same significant effect on chlorophyll synthesis related genes, which was statistically significantly different from other interactions. Interestingly, MgONPs at 200 μg/mL had the most significant effect on photosystem structure genes. Mg is an important element for photoassimilation and photophosphorylation. Tamil Elakkiya et al. (2020) reported that the application of MgONPs increased chlorophyll content by six times. Therefore, the increase in the chlorophyll synthesis related genes as a result of nanoparticle application in this investigation could explain the increment observed in the photosynthetic apparatus of Arabidopsis, rice growth parameters, and biomass.

The significant bacterial disease suppression by the application of nanoparticles in this study is consistent with (Cai et al., 2018) studies on bacterial wilt disease reduction in tobacco plants when amended with MgONPs. Cai et al. (2018) reported an increase in the height and weight of tobacco plants after amendment with MgONPs. Therefore, the application of MgONPs and MnO_2_NPs on rice plants in this study increased its growth parameters and biomass and reduced the disease expression of BLB. Interestingly, MnO_2_NPs were discovered to perform better than MgONPs in growth improvement in rice plants. This could be due to the function of Mn as an important micronutrient for plant growth. Hence, this result is in agreement with Elmer and White (2016), who reported a significant improvement in the growth of wilt-diseased tomato plants when sprayed with nanoparticles of AlO, ZnO, FeO, CuO, and MnO.

Exopolysaccharide (EPS) production and biofilm development are key contributing factors to the virulence pathogenesis in Xoo as they are essential for bacterial colonization in xylem vessels and symptom expression (Kim et al., 2009; Rai et al., 2012). Zhang et al. (2013), in an earlier study, reported a high dependency between biofilm formation and virulence. Therefore, to determine the efficacy of MgONPs and MnO_2_NPs as good bio-control agents, their antibiofilm effect on Xoo is important. In summary, by two-way ANOVA analysis, it can be concluded that the efficient antibiofilm activity observed with the application of MgONPs and MnO_2_NPs at 200 μg/mL was significantly different from other interactions, which correlated with the reduction of BLB disease expression.

The entry of nanoparticles into bacterial cells, invariably causing cell structure deformation, is documented as the fundamental mechanism for bacterial inhibition (Raghupathi et al., 2011). Furthermore, the loss of cellular materials as a result of nanoparticle treatment is one of the mechanisms used in antibacterial activity since much of the bacterial activity depends on the cellular content (Ananda et al., 2018). Notably, MgONPs at 200 μg/mL had the most statistically significant effect on cellular efflux when compared to other interactions. Flow cytometry and PI staining fluorescence microscopy, which only stains dead or injured cells, were used to further substantiate the mechanism of action of MgONPs and MnO_2_NPs. It is pertinent to note that exposure of bacterial cells to metal oxide nanoparticles has been reported to cause an increase in cell apoptosis (Cai et al., 2018; Das et al., 2019; Chakraborty et al., 2023).

## 5. Conclusion

MgONPs and MnO_2_NPs were produced using phage X3 lysate. The success of the synthetic protocol used was confirmed with the observation of an absorption peak at 215 nm for MgONPs, while the MnO_2_NPs peak was observed at 230 nm. The produced MgONPs and MnO_2_NPs efficiently reduced Xoo virulence by inhibiting its growth and biofilm formation at a concentration of 200 μg/mL. This distinct characteristic of MgONPs and MnO_2_NPs was reflected in the reduction of bacterial leaf blight expression on rice plants *in vivo.* However, MnO_2_NPs were found to significantly improve the growth parameters of rice plants compared to MgONPs. Using phage X3 lysate to produce MgONPs and MnO_2_NPs was found to be ecologically friendly as it had no adverse effects on the chlorophyll fluorescence parameters when tested on Arabidopsis. Our research depicts the use of nanoparticles, paving the way for the development of novel crop protection products in the near future. Both MgONPs and MnO_2_NPs at 200 μg/mL showed the most significant impact. MgONPs at 200 μg/mL revealed a significant impact on photosystem structure genes, which resulted in the efficacy of the photosynthesis process. MnO_2_NPs were found to have the most significant effect on antagonism on NA plates, while MgONPs had the most significant impact on the bacteria growth in NB and on cellular efflux. Finally, there is no doubt that nanoparticles will play a vital role in the improvement of plant disease management, as it is suitable for use in varied agricultural products that safeguard plants from bacterial blight disease and oversees plant growth.

## Data availability statement

The original contributions presented in the study are included in the article/supplementary material, further inquiries can be directed to the corresponding authors.

## Author contributions

SO and YA: conceptualization, methodology. EI, YZ, and SO: software. SO, YA, and EI: validation. TA and SO: formal analysis. YZ: investigation. DA, WH, BL, and CY: resources. JB and SO: data curation. SO: writing—original draft preparation. DA, WH, and TA: writing—review and editing. JB, LX, CY, and YA: visualization. CY, LX, and BL: supervision. BL: funding acquisition. All authors contributed to the article and approved the submitted version.

## Funding

The work was partially supported by the National Key Research and Development Program of Ningbo (2022Z175, 2019B10004), the Key Research and Development Program of Zhejiang Province, China (2019C02006), the Zhejiang Provincial Natural Science Foundation of China (LZ19C140002), the State Key Laboratory for Managing Biotic and Chemical Threats to the Quality and Safety of Agro-Products (grant numbers 2010DS700124-ZZ2014, 2010DS700124-KF202101, and 2010DS700124-KF202205), and Princess Nourah Bint Abdulrahman University Researchers Supporting Project number (PNURSP2023R15).

## Conflict of interest

The authors declare that the research was conducted in the absence of any commercial or financial relationships that could be construed as a potential conflict of interest.

## Publisher’s note

All claims expressed in this article are solely those of the authors and do not necessarily represent those of their affiliated organizations, or those of the publisher, the editors and the reviewers. Any product that may be evaluated in this article, or claim that may be made by its manufacturer, is not guaranteed or endorsed by the publisher.
